# Clinical manifestations and endoscopic findings of amebic colitis in a United States-Mexico border city: a case series

**DOI:** 10.1186/s13104-015-1787-3

**Published:** 2015-12-14

**Authors:** Rhonda Fleming, Chad J. Cooper, Ruben Ramirez-Vega, Ana Huerta-Alardin, Darius Boman, Marc J. Zuckerman

**Affiliations:** Department of Internal Medicine, Texas Tech University Health Sciences Center, 4800 Alberta Ave, El Paso, TX 79905 USA; Department of Pathology, Texas Tech University Health Sciences Center, 4800 Alberta Ave, El Paso, TX 79905 USA

**Keywords:** Amebic colitis, Amebiasis, Diarrhea, Hematochezia, Colonoscopy

## Abstract

**Background:**

Invasive amebiasis is not frequently seen in the United States. It is associated with considerable morbidity in patients residing in or traveling to endemic areas. We report a case series of patients with amebic colitis in a United States-Mexico border city to alert physicians to the varied clinical manifestations.

**Case presentation:**

Nine patients were diagnosed with amebic colitis. Mean age was 56 (38–83), 6 were males, and all were Hispanic. Common symptoms were diarrhea (56 %), hematochezia (33 %) and abdominal bloating (11 %). The diagnosis of amebic colitis was established in the following ways: 8 patients by colonoscopy with biopsy, 1 by surgery for colonic obstruction. The diagnosis of amebic colitis was confirmed in 8 patients (89 %) by amebic trophozoites present in histopathologic sections. One patient was diagnosed with amebic colitis based upon clinical symptoms, colitis on colonoscopy and visualization of amebic trophozoites on stool examination. In the 8 patients in whom colonoscopy was done, 6 (75 %) had inflammation with rectosigmoid involvement and 5 (62.5 %) had ulcerations. Infection resolved after treatment with metronidazole in most patients; however, one patient developed a liver abscess and another had a colonic perforation and later developed a liver abscess.

**Conclusion:**

The occurrence of amebic colitis in this United States-Mexico border city hospital population was low, but in some cases potentially life-threatening. Physicians should be alert to the less common presentations of amebic colitis, such as overt gastrointestinal bleeding, exacerbation of inflammatory bowel disease, and the incidental finding of association with colon cancer, or a surgical abdomen. Rectosigmoid involvement was typically found on colonoscopy.

## Background

*Entameba histolytica* infects 10 % of the world’s population, resulting in 50 million cases of invasive amebiasis (colitis and liver abscess) and 100,000 deaths annually [1–6]. In developed countries such as the United States, amebiasis is most often seen in recent immigrants, travelers to an endemic area, homosexuals, institutionalized or immunocompromised individuals [7, 8]. Amebic colitis may present with fever, abdominal pain, watery or bloody diarrhea [9]. The diagnosis of amebic colitis can be difficult because the gastrointestinal symptoms are nonspecific and can mimic other colonic diseases. Complications of amebic colitis include fulminant amebic colitis, intestinal perforation, peritonitis, hemorrhage, strictures or obstruction [10–15]. Amebic liver abscesses occur in 3–9 % of all cases of intestinal amebiasis [16, 17]. Some individuals presenting with amebic liver abscess have concurrent amebic colitis, but more often they have no bowel symptoms and stool microscopy is negative for *E. histolytica* trophozoites and cysts. Ameboma is a rare complication that is found in the cecum or ascending colon [18]. The most reliable method for diagnosing intestinal amebiasis is the demonstration of the trophozoites on tissue biopsy [19–22].

We have observed the presence of amebiasis on the United States-Mexico border, but it is not common. In fact, in a review of 2744 stool specimens submitted to our hospital’s lab over a 2 year period. *E. histolytica/E. dispar* was only found in 6 (0.22 %) of stool specimens [23]. We present a case series of patients with amebic colitis in a United States-Mexico border city to alert physicians to this potentially life-threatening disease and to present its various clinical manifestations and endoscopic findings.

## Methods

This study is a retrospective chart review that was performed on all patients diagnosed with clinical and endoscopic manifestations of amebic colitis between the period of October 1, 1999 through April 30, 2009 at Texas Tech University Health Sciences Center of El Paso, Texas and its affiliated hospital, University Medical Center of El Paso. The study protocol was approved by the Institutional Review Board (IRB) of Texas Tech University Health Sciences Center of El Paso, Texas.

The following criteria were used to establish the diagnosis of amebic colitis: (1) history of bloody diarrhea; (2) endoscopic evidence of diffuse mucosal inflammation, with or without mucosal ulceration; (3) presence of motile hematophagous trophozoites of *E.histolytica* in colon biopsy or stool samples; and (4) histologic confirmation of invasive amebiasis in colonic tissue. The presence of cysts of *E. histolytica* or a positive indirect hemagglutination test for amebiasis was not criteria for diagnosis of amebic colitis. Symptomatic patients with *E. histolytica* in stool who also demonstrated pathogenic virus or bacteria in stool sample or colonic tissue were excluded from the study. The demographic, clinical manifestations, physical examination and laboratory findings, endoscopic and pathology reports and management were analyzed retrospectively.

### Statistical analysis

Descriptive statistical analysis are presented as mean, median or percentage for variables measured on a continuous scale (e.g., age) and as proportions for variables measured on a discrete scale (e.g., gender).

### Case presentation

Nine patients were diagnosed with amebic colitis with a mean age of 56 (range 38–83), 6 were male, and all were Hispanic. Clinical manifestations are described in Table 1. None of the patients reported previous episodes of dysentery or amebiasis. The main symptoms were diarrhea (56 %), hematochezia (33 %) or abdominal distension (11 %). Secondary complaints included fever, anemia, abdominal pain and rectal discomfort/pain. Median duration of symptoms was from 5–14 days; one patient had chronic episodes of hematochezia lasting approximately 6 months. Patient 6 had recurrent bouts of chronic diarrhea likely related to flares in her ulcerative colitis. Physical findings included abdominal distention (33 %), abdominal tenderness to palpation (67 %) that was either localized (33 %) or diffuse (67 %). Other abdominal examination findings included decreased bowel sounds or a normal exam. One patient was found to have a palpable mass in the right lower quadrant (RLQ) that was first thought to be due to a partial small bowel obstruction, but this was later discovered to be from a large cecal mass.

**Table 1:** Overall characteristics of patients with amebic colitis

No.	Sex	Age	Main symptom	Secondary symptom	Physical findings	Duration of symptoms (days)	Diagnosis (amebic trophozoites)
1	F	45	Abdominal distension	Emesis	Abdominal distention, RLQ tenderness with a palpable mass	5	Colon biopsy (+)
2	F	83	Diarrhea	Abdominal pain	Abdominal distension, decreased bowel sounds	14	Colon biopsy (+)
3	M	38	Diarrhea	Fever	Diffuse tenderness to palpation	7	Colon biopsy: Non-diagnostic Stool (+)
4	M	46	Diarrhea	–	Abdominal distention, diffuse tenderness	14	Colon biopsy (+)
5	M	70	Diarrhea	Abdominal pain	Tender to palpation LLQ	10	Colon biopsy (+) Stool (+)
6	F	60	Diarrhea	Abdominal pain	Diffuse abdominal tenderness	14	Colon biopsy (+)
7	M	45	Hematochezia	Rectal pain	Normal abdominal exam	14	Colon biopsy (+)
8	M	44	Hematochezia	Rectal discomfort	Diffuse abdominal tenderness	7	Colon biopsy (+) Stool (+)
9	M	76	Hematochezia	Anemia	Normal abdominal exam	180	Colon biopsy (+)

*RLQ* Right lower quadrant, *LLQ* left lower quadrant, *NS* None stated

The diagnosis of amebic colitis was established in the following ways: 8 patients by colonoscopy with biopsy and 1 by surgical histopathology analysis after undergoing an exploratory laparotomy with a right hemicolectomy for colonic obstruction. In the 8 patients that had a colonoscopy, the cecum/ileum was reached in 4 patients. Endoscopic, histopathological and surgical findings are in Table 2. All 8 patients who underwent colonoscopy had various abnormal endoscopic findings. Inflammation of the colon with or without ulceration was the most frequently encountered endoscopic finding. 6 of 8 patients (75 %) in whom colonoscopy was done had inflammation with rectosigmoid involvement and 5 (62.5 %) had ulcerations.

**Table 2:** Endoscopic, surgical findings and outcome of patients with amebic colitis

No.	Procedure	Location/distance	Findings	Histopathology	Treatment	Outcome
1	Surgery	Terminal ileum, cecum, ascending colon	Large cecal mass	Extensive flask shaped ulcers with large abscesses with numerous hemophagocytic amebic trophozoites	metronidazole 14 days	5 days later: colonic perforation secondary to amebiasis. Ileostomy. 1 year later: amebic liver abscess
2	Colonoscopy	To transverse	Multiple ulcers	Amebiasis with deep ulcerations	metronidazole 14 days + paromomycin 7 days	Complete resolution of symptoms
3	Colonoscopy	To splenic flexure	Rectosigmoid ulceration/colitis	Acute and chronic colitis. No amebic trophozoits found	metronidazole 14 days + paromomycin 10 days	7 days later: amebic liver abscess
4	Colonoscopy	To descending	Severe rectosigmoid colitis/ulcerations	Surface ulcerations covered by necrotic fibrinous exudate. Many amebic trophozoites in exudate	metronidazole 14 days + paromomycin 20 days	Complete resolution of symptoms
5	Colonoscopy	To ileum	Pancolitis/multiple ulcers	Mucosal edema and ulcerations/necrosis with fibrinous exudate. Hemophagocytic amebic trophozoites in exudate	metronidazole 14 days	Discharged in good condition.
6	Colonoscopy	To cecum	Diffuse inflammation/pseudopolyps	Acute and chronic colitis with superficial erosion/necrosis. Amebic trophozoites present	metronidazole 14 days + paromomycin 10 days	Amebiasis resolved. Diarrhea persists due to ulcerative colitis
7	Colonoscopy	To ileum	Pancolitis/multiple ulcers	Acute colitis. Amebic trophozoites present	metronidazole 14 days + iodoquinol 20 days	Discharged in good condition.
8	Colonoscopy	To cecum	Pancolitis	Acute and focal colitis. Amebic trophozoites present	metronidazole 14 days	Resolution of symptoms
9	Colonoscopy	To hepatic flexure	Fungating, ulcerated, partially obstructing mass in distal sigmoid colon	Invasive Sigmoid Adenocarcinoma. Amebic trophozoites present	metronidazole 14 days + paromomycin 7 days	Chemotherapy

*RLQ* right lower quadrant, *LLQ* left lower quadrant, *ND* not done, *NS* not stated

For clinical reasons, the cecum was not reached in all colonoscopies. Involvement of the cecum and ascending was observed in 50 % of cases (three cases of pancolitis and one case of cecum and ascending colon) and 50 % of these had ulcerations present. The diagnosis of amebic colitis was confirmed in 8 (89 %) patients in whom amebic trophozoites were present in histopathologic sections. Only one case (Patient 3) with rectosigmoid ulcerations, had no evidence of invasive amebiasis in histopathologic examination, but stool examination revealed the presence of amebic trophozoites. One patient had concomitant inflammatory bowel disease (ulcerative colitis) and the diagnosis of amebic colitis was made based on *E. histolytica* serology and histological examination of biopsies from colonoscopy. One (11 %) patient had amebiasis associated with invasive sigmoid adenocarcinoma. The amebic colitis infection resolved after treatment with metronidazole usually followed by a course of paromomycin or iodoquinol in 6 (67 %) patients. However, 2 (22 %) patients developed an amebic liver abscess.

### Case summaries

#### Case 1

A 45 year old Hispanic woman presented with fever, vomiting and abdominal distention for 5 days. Vital signs revealed hypertension and tachycardia. Physical examination revealed abdominal distention and tenderness with a palpable mass in the right lower quadrant. The abdominal CT on admission revealed a partial small bowel obstruction and a right colonic mass. She underwent an exploratory laparotomy with a right hemicolectomy with ileotransverse colon anastomosis. Pathological findings discovered extensive amebic colitis with perforation, necrosis, acute and chronic inflammation with abscess formation and peritonitis in the terminal ileum and right colon. Microscopic examination of the terminal ileum and right colon revealed multiple flask shaped ulcers and numerous *E. histolytica* trophozoites. One week later she developed peritonitis secondary to an anastomotic leak that required another exploratory laparotomy with resection of the ileotransverse anastomosis and creation of an ileostomy. She was subsequently discharged on metronidazole therapy. Five months later she was readmitted for ileostomy takedown and ileocolonic anastomosis. Nine months after her first admission, she presented complained of diarrhea and hematochezia for 6 months. An upper endoscopy found an extraluminal mass close to the fundus of the stomach. Abdominal CT revealed an amebic liver abscess. She was treated with metronidazole for 2 weeks followed by paromomycin for 1 week.

#### Case 2

An 83 year old Hispanic woman presented with weakness, diarrhea, abdominal cramps, tenesmus for 2 weeks. Her concurrent medical problems included hypertension, diabetes and a stroke. Vital signs revealed hypotension. Significant physical examination included abdominal distention and hypoactive bowel sounds. Laboratory findings were significant for anemia, leukocytosis with a bandemia, hyponatremia and hyperglycemia. A colonoscopy was done 2 days later and revealed multiple ulcers throughout the entire colon and rectum. Histopathology of the biopsy specimens revealed amebiasis with deep ulcerations in the distal transverse, proximal descending and rectum. She was treated with metronidazole for 2 weeks followed by paromomycin for 1 week.

#### Case 3

A 38 year old Hispanic man presented with fever, chills, weakness, nausea, vomiting, loss of appetite and watery diarrhea for 1 week. His concurrent medical problems included hepatitis C. Vital signs revealed a high grade fever and tachycardia. Physical examination was significant for diffuse abdominal tenderness. Laboratory findings were significant for leukocytosis and hyponatremia. Stool studies revealed *E. histolytica* trophozoites. Five days after admission, a colonoscopy revealed rectosigmoid ulcerations with histopathology showing acute and chronic colitis. He was discharged on a 10 day course of metronidazole and paromomycin. Seven days after being discharged, the patient was readmitted for right upper quadrant pain and shortness of breath. Abdominal CT revealed two large liver abscesses that were subsequently drained by interventional radiology and found to be positive for *E. histolytica*. He was subsequently discharged and retreated with a 10 day course of metronidazole followed by paromomycin for another 10 days.

#### Case 4

**Fig. 1:**
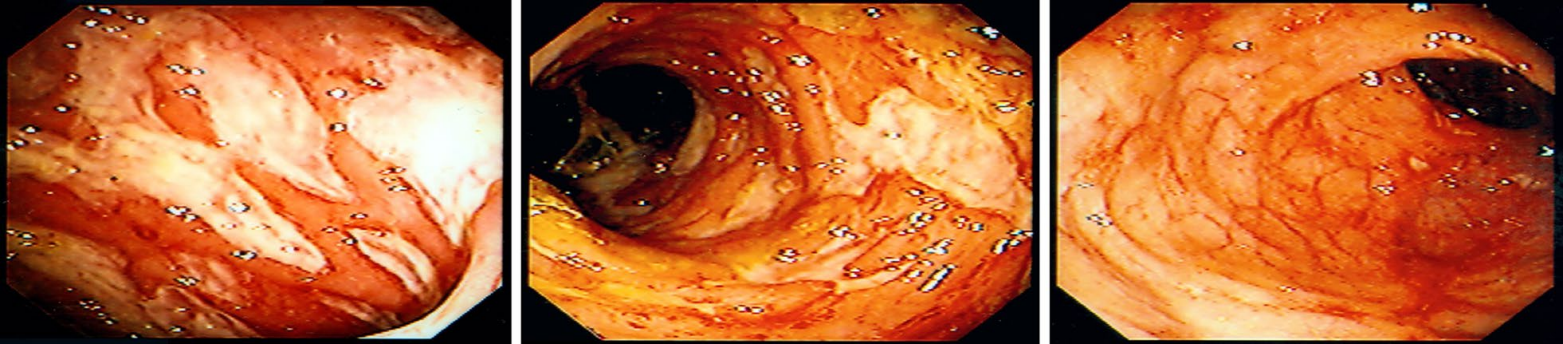
Endoscopic findings of a patient who presented with diarrhea

**Fig. 2:**
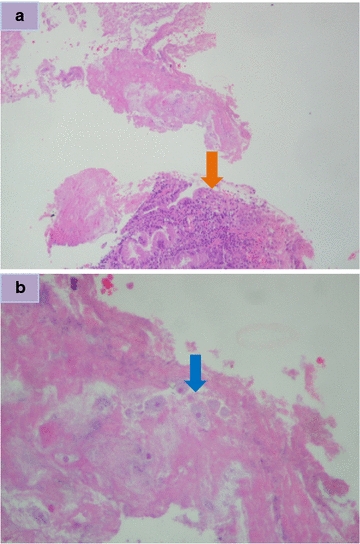
**a** Low power microscopic view (100×) of biopsy from same patient showing an inflammatory exudate containing amebas floating free close to the surface colonic mucosa (*orange arrow*). **b** High power microscopic view (400×) of biopsy from same patient of the mucosal exudate displaying amebas, with a red cell in their cytoplasm (*blue arrow*)

A 46 year old Hispanic man presented with watery diarrhea for 1 month that became bloody within the last 2 weeks. Vital signs revealed tachycardia. Physical examination findings were significant for abdominal distention and diffuse tenderness to palpation and guarding. Laboratory findings were significant for leukocytosis, anemia, hyponatremia, hypokalemia and hyperglycemia. Stool studies revealed *E. histolytica* trophozoites. One day after admission, a colonoscopy revealed rectosigmoid colitis and ulceration with histopathology revealing surface ulcerations covered by fibrinous exudate containing hemophagocytic amebic trophozoites (Figs. 1, 2a, b). He was subsequently discharged with a 2 week course of metronidazole followed by paromomycin for another 20 days.

#### Case 5

A 70 year old Hispanic man presented with abdominal pain and watery diarrhea for 10 days. Concurrent medical problems included diabetes mellitus. Physical examination findings included abdominal tenderness to palpation at the left lower quadrant. Laboratory findings were significant for leukocytosis with a bandemia, anemia, hyponatremia and hyperglycemia. Stool studies revealed *E. histolytica* trophozoites. Amebic serology was positive. One day after admission, a colonoscopy revealed pancolitis with multiple ulcerations throughout the entire colon with histopathology revealing mucosal edema, ulcerations and necrosis with fibrinous exudate containing hemophagocytic amebic trophozoites. He was subsequently discharged with a 2 week course of metronidazole.

#### Case 6

A 60 year old Hispanic woman presented with watery diarrhea and abdominal pain for 2 weeks. Concurrent medical problems included ulcerative colitis for more than 20 years, currently being treated with prednisone and sulfasalazine. Physical examination findings included diffuse abdominal tenderness. Laboratory findings were significant for leukocytosis and anemia. Stool studies revealed *E. histolytica* trophozoites. Amebic serology was positive. One day after admission, a colonoscopy revealed diffuse inflammation and multiple pseudopolyps. Histopathologic examination was consistent with acute and chronic colitis with superficial erosions, focal liquefactive necrosis and amebic trophozoites. The patient was subsequently discharged with a 2 week course of metronidazole followed by 10 days of paromomycin.

#### Case 7

**Fig. 3:**
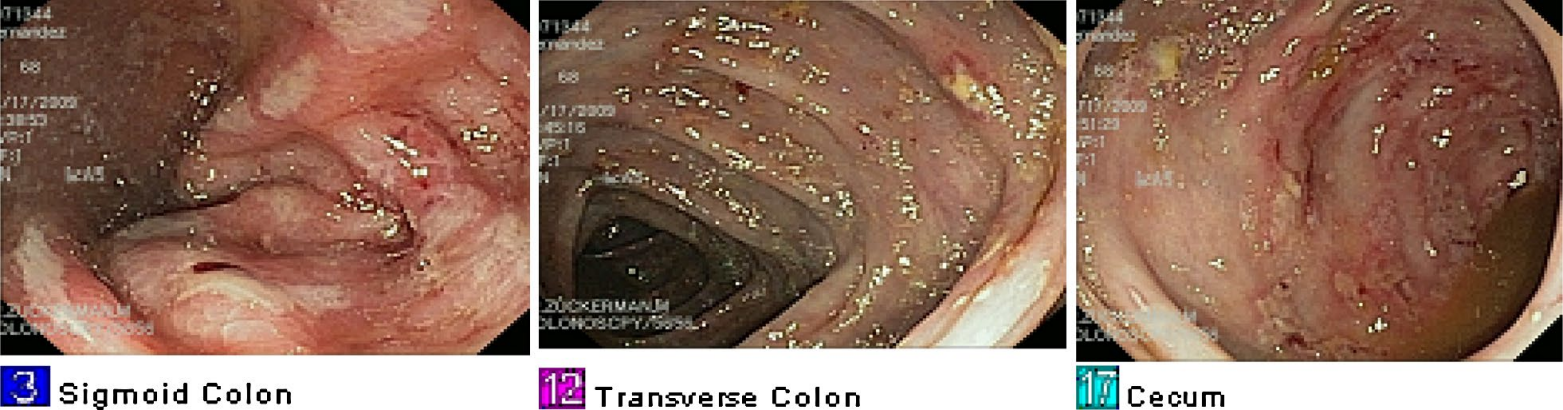
Endoscopic findings of patient who originally presented with hematochezia

**Fig. 4:**
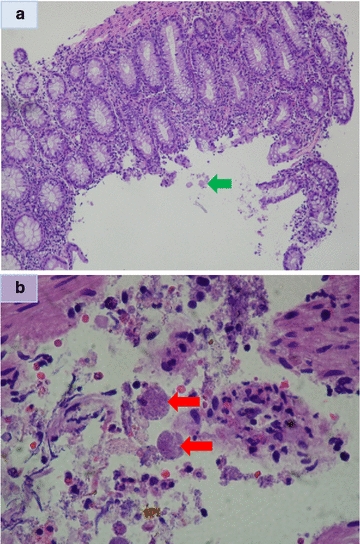
**a** Low power microscopic view (100×) of biopsy from same patient showing an inflamed colonic mucosa with preservation of its architecture and amebas floating free close to the surface epithelium (*green arrow*). **b** High power view (400×) of biopsy from same patient of the mucosal exudate displaying two amebas, one of them with a red cell in its cytoplasm (*red arrow*)

A 45 year old Hispanic man presented with hematochezia and rectal pain for 2 weeks. Vital signs were significant for hypotension. Physical examination findings of the abdomen were unremarkable. However, rectal examination was positive for occult blood but no masses were felt in the rectal vault. Laboratory findings were significant for leukocytosis, severe anemia, hyponatremia and hypokalemia. One day after admission, a colonoscopy revealed pancolitis with severe proctitis and perirectal ulcerations (Fig. 3). Histopathologic examination was consistent with acute colitis with amebic trophozoites (Fig. 4a, b). The patient was subsequently discharged with a 2 week course of metronidazole followed by 20 days of iodoquinol.

#### Case 8

A 44 year old Hispanic man presented with diarrhea for 7 days prior to admission followed by constipation, rectal discomfort, and melena. Past medical history was significant for hepatitis C infection. Physical examination findings included diffuse abdominal tenderness. Lab results were significant for elevated transaminases and total serum bilirubin of 1.2 mg/dL. Stool examination was positive for *Clostridium difficile* toxin. An abdominal and pelvic CT scan showed thickening of the rectal wall. A subsequent colonoscopy showed a normal terminal ileum, with congested, erythematous, eroded, granular mucosa throughout the colon and rectum. No areas of ulceration or erosions were present. Biopsies revealed focal active colitis and microorganisms compatible with *E. histolytica.* Amebic serology was positive. He was treated with metronidazole for 14 days, for *C. difficile* infection and invasive amebiasis.

#### Case 9

A 76 year old Hispanic man presented with intermittent hematochezia for 6 months and a 20 pound weight loss. Physical examination of the abdomen was unremarkable. Lab results were significant for anemia. Abdominal CT showed soft tissue densities of the sigmoid colon with extra colonic invasion and associated perilesional adenopathy and liver metastases. A subsequent colonoscopy revealed two fungating and ulcerated partially obstructive masses in the distal sigmoid colon and distal descending colon. Pathological findings confirmed a sigmoid adenocarcinoma, with positive amoeba on PAS stain. Liver biopsy showed metastatic poorly differentiated adenocarcinoma. An amebic serology was positive at a titer of 1:256. Due to persistent hematochezia, a sigmoid resection followed by formation of a colostomy was performed. Pathology of the sigmoid colon mass from the segmental colectomy showed invasive adenocarcinoma of the sigmoid colon, with the tumor invading the entire thickness of the colonic wall, pericolic soft tissue, and directly invading an adjacent segment of colon with direct extension into the mucosa forming a secondary mucosal ulcerated mass. The original ulcerated tumor bed was invaded by trophozoites of *E. histolytica* with organisms present in the necrotic and inflammatory debris and infiltrating the tumor parenchyma. In our case this was an adenocarcinoma infiltrated by amebic trophozoites and not an ameboma. Treatment was with metronidazole IV for 14 days followed by paromomycin for 7 days.

## Conclusions

The clinical manifestations of amebic colitis are varied, as demonstrated in our case series of amebic colitis. In our patients, the most common manifestations were diarrhea or hematochezia. Less common presentations included a patient with an inflammatory bowel disease flare with superimposed amebic colitis, bowel perforation and amebic trophozoite invasion of a colon adenocarcinoma. Inflammation of the colon, with or without ulceration, was the most frequently encountered endoscopic finding. The typical method for diagnosing intestinal amebiasis was the histopathological demonstration of the trophozoites in the biopsied lesions. Treatment of amebic colitis with metronidazole with or without paromomycin was successful in the majority of patients but complications such as amebic liver abscess did occur.

There have been a few worldwide case series or studies that report on the various clinical manifestations of amebic colitis [11, 17, 24–30]. The classic study from South Africa by Adams in 1977 was an analysis of 3013 adult patients admitted with intestinal amebiasis [12]. The majority of patients presented with watery diarrhea, followed by bloody diarrhea and abdominal pain for at least 1–4 weeks. These symptoms were also observed in other clinical studies [1, 24–27]. These retrospective studies observed less common complaints of high grade fever, severe weight loss, tenemus and overt gastrointestinal bleeding [24, 27]. The most frequent physical examination findings in these studies were abdominal tenderness, tachycardia, hypotension and abdominal rebound [27]. Common laboratory findings were anemia, and leukocytosis, which was also observed in our case series [1, 25, 27]. Patients with amebic liver abscess may demonstrate an elevation of liver function tests and erythrocyte sedimentation rate and/or other acute phase reactants [28].

Colonoscopy combined with biopsy or intestinal fluid sample collection can be useful in distinguishing amebic colitis from other colonic entities. The mucosal appearance of amebic colitis on endoscopy may be grossly indistinguishable from that of intestinal tuberculosis, other forms of infectious colitis and inflammatory bowel disease [29]. Common but nonspecific endoscopic findings include diffusely inflamed and friable mucosa [25, 26]. Histological examination will usually demonstrate ulcerations, acute and/or chronic inflammation with amebic trophozoites invading through the bowel wall [27].

We would like to highlight four important cases in the series, including case 1—bowel perforation with formation of liver abscess, case 3—liver abscess formation, case 6—ulcerative colitis exacerbation and case 9—adenocarcinoma with amebic infiltration. We cannot speculate on the relationship of patients suffering from Hepatitis C followed by the burden of amebic colitis and formation of amebic liver abscess. A few studies have evaluated the predictive value of the location of endoscopic findings in the diagnosis of acute intestinal amebiasis. These studies concluded that the cecum is the most common site involved in amebic colitis [17, 29, 30]. Other studies have evaluated the endoscopic findings in patients with amebic colitis and an amebic liver abscess. In a prospective study from India, colonic lesions in patients with amebic liver abscess most commonly involved the cecum followed by the ascending colon [30]. However, in their control group of patients with only amebic colitis, they found more extensive disease with large ulcers and diffuse mucosal inflammation in the left colon, predominantly the rectosigmoid and descending colon [30].

One of our patients presented with a flare of inflammatory bowel disease. This has also been described in another case report [31]. A study from Turkey that found *E. histolytica/E. dispar* cysts and trophozoites in 14 (8.75 %) of 160 cases, 13 (10 %) of 130 patients with ulcerative colitis and 1 (3.3 %) of the 30 patients with Crohn’s disease [32]. Amebic infection in these patients with inflammatory bowel disease was more prevalent than in the normal population [32]. A study from Bosnia–Herzegovina analyzed 31 patients with ulcerative colitis and co-infected with ameba. They concluded that ameba infection in ulcerative colitis patients was not related to the grade of disease activity and other clinical variables such as gender, age and parameters of inflammation [33].

We have one patient in our case series with the rare occurrence of amebic trophozoite invasion of colonic adenocarcinoma. A couple of case series have found the association of amebiasis with adenocarcinoma. They observed that the amebic trophozoites invaded the colorectal adenocarcinoma, but invasion was not present in the normal colonic mucosa [34, 35]. Invasion of the amebic trophozoites into the tumor appeared to be coincidental, rather than the etiology of the adenocarcinoma [34, 35]. The disruption in the mucosa and reduced efficiency of local immune mechanisms caused by the carcinoma may have facilitated invasion of the trophozoites through the mucosa [35]. A case report from India described a patient with clinical and radiological suspicion of colonic carcinoma. However, after resection of perforated cecum, histopathological examination showed numerous trophozoites of *E. histolytica* in a background of abundant necrosis [36]. This case suggests that the differentiation between ameboma and carcinoma is critical.

Our study has some limitations. The number of cases seen in this time period was modest and may have been subject to bias. Not all patients with symptoms were required to have a colonoscopy. This was a respective study and every patient reported had a colonoscopy but the endoscopist decided how far they needed to go during colonoscopy based on clinical judgement.

Not every patient had a complete colonoscopy that reached the terminal ileum, therefore we cannot comment on the right colon in some patients. A real-time PCR assay targeting *E. histolytica* specific probe was not utilized in our cases to confirm our results, as has been reported in Bangladesh [37]. It has the strengths of describing cases in detail of amebic colitis in the Hispanic population of the United States-Mexico border.

In conclusion, the occurrence of amebic colitis in our United States-Mexico border city hospital population was not common, but some cases were potentially life threatening. Clinicians should be alert to the varied clinical manifestations and endoscopic findings of amebic colitis. The most common symptoms in our patients were diarrhea and hematochezia, but physicians should be alert to the less common presentations of overt gastrointestinal bleeding, inflammatory bowel disease flares, acute abdomen or association with colon cancer. Rectosigmoid involvement was typically found on colonoscopy; however, other case series have reported that the cecum is the most commonly involved site. Endoscopic evaluation should be utilized to assist in the diagnosis, with attention to the observation of colonic inflammation, ulceration and amebic trophozoites on histopathological examination.

## Consent

Written informed consent was obtained from the patients for publication of this case report and any accompanying images.

## Abbreviations

IRB: Institutional Review Board
CT: computed tomography
RLQ: right lower quandrant
PAS: periodic acid–Schiff
